# Effectiveness of digital education shifting model on high school students’ engagement

**DOI:** 10.1007/s10639-021-10879-4

**Published:** 2022-01-27

**Authors:** Fayiz M. Aldhafeeri, Asmaa A. Alotaibi

**Affiliations:** Kuwait University, Kuwait City, Kuwait

**Keywords:** Digital Education Shifting, Student Engagement, Digital Learning, Remote Learning, Distance Education, Learning Effectiveness

## Abstract

Digital learning has often been criticized due to its lack of student engagement, which results from the physical absence of teachers and the lack of direct communication with their students. Critics of digital education have also pointed out that students’ disengagement is a major factor behind this education format’s inability to positively impact all stakeholders. This can be frustrating for the students themselves and may result in poor educational outcomes. Therefore, digital learning is regarded as a temporary arrangement and not a potential replacement for face-to-face education because of shortcomings that can lead to disengagement among students. To test the validity of this belief, we conducted an experimental study on 245 female Kuwaiti students. We trained teachers in the digital education shifting (DES) model. Following this model, we placed emphasis on student–teacher communication, cooperation among students, and enhanced the principles of digital learning. We asked the trained teachers’ students to respond to a self-assessed student engagement checklist survey to measure their engagement during online classes. We found that the students of the experimental group performed better on various parameters of observable and internal engagement compared to control group students. Contrary to general belief, we proved that the innovative DES approach can indeed make digital learning more engaging, effective, and a viable alternative or at least an aligned and integrated form for conventional education in the long run.

## Rationale of the study

The internet has penetrated all aspects of our lives because all industries have steadily developed significant reliance on it. The education industry is no exception; the internet introduced online learning, which made education more accessible to people than ever before. In an era characterized by rapid growth and development of information technology, the internet offers a convenient two-way interactive session for learning that is not confined to a classroom. Various platforms provide basic and more advanced learning opportunities to those seeking information and knowledge. The inclusion of computerized information in learning instruction can spark students’ interest in coursework, significantly enhancing study outcomes. The rapid growth of network and computer applications in the 1990s created a breakthrough in distance learning that does not restrict a student to one physical location and can achieve the same desired outcomes as the classroom (Harasim, 2000). Thus, the internet has also allowed new pedagogical models and approaches to emerge. Merging technological resources with creative educative approaches has transformed the teaching and learning processes significantly.

Before the development of the internet, remote learning was available for students who were interested in gaining specific skills or learning about particular subjects. In the 1840s, Isaac Pitman taught his pupils using correspondence with the aim of improving writing speed (Archibald & Worsley, 2019). The pupils would send complete class assignments to Pitman by mail. In 1954, B.F. Skinner designed a machine that gave schools the ability to give students programmed instructions. In 1960, the first computer-based training program; Programmed Logic for Automated Teaching Operations (PLATO) was introduced (Archibald & Worsley, 2019). In 1969, ARPANET was introduced, which featured the basic concepts of the internet, but the technology was not made available to the public until 1989 (Harasim, 2000). In the late twentieth century, the introduction of personal computers and the internet paved way for better e-learning and teaching delivery approaches. However, the first fully online course was not offered until 1981, and the adoption of online learning was slow: the first online degree program was initiated in 1986 (Harasim, 2000).

Since their inauguration in the early 1990s, web-based courses have become common at all education levels, to the point that the 2011 Sloan Survey on Online Learning showed that the number of students taking at least one online course had surpassed 6 million, which was nearly 30% of all students in higher education (Silverstone & Keeler, 2013; Swann, 2013). Unfortunately, most research on the issue of online education has focused on higher education and ignored K–12 education. Part of the reason for this is that online learning at the higher education level was introduced to solve the issue of accessibility to college education.

According to the National Center for Education Statistics’ Integrated Postsecondary Education Data System, in 2018, about 35.3% of students were attending distance learning courses under degree programs (National Center for Education Statistics, n.d.). Private institutions saw a high remote learning enrollment rate of 73%, but public institutions had only 34.1% of students enrolled in the same types of programs. This trend changed in 2020, when the COVID-19 pandemic struck and forced almost every higher education learning center to change its mode of training to distance learning (Pelikan et al., 2021). Students had to find the motivation to take part in distance learning and modify their plans to fit in their learning at home. According to the U.S. Department of Education (n.d**.**), more than 48 states adopted information and communications technology in education at several levels. Some distance learning programs fall under the management of states, and others under individual counties. There are several developers of such technology across the nation.

With the arrival of technological advancements, online learning continued to gain ground and has become the preferred option among many learners (Putman et al., 2012). The challenge that scholars and education practitioners continue to face in this fast-track equation is that they need to ensure that learners get the best out of their online education experiences. As opposed to traditional learning, where students have the opportunity to meet in person, online environments do not possess such a feature. Therefore, researchers have attempted to design and produce effective methods of engaging students in online courses (e.g., Khan et al., 2017; Metcalf & Haugen, 2018; Powers, 2020).

### Problem statement

Numerous attempts have been made to introduce some kind of change to education, especially in terms of technology adoption in the classroom, but such attempts have not yielded desirable results (McQuirter, 2020; Zhao & Watterston, 2021). Zhao and Watterston (2021) stated that education must undergo profound change and that all stakeholders should seriously consider reimagining education. The paradox around education is that even though everything else in our lives has gone through substantial changes, education is stagnant. For the past 100 years or so, education has failed to prepare learners for the modern world, let alone for the future (Barber et al., 2012; Wagner, 2008; Wagner & Dintersmith, 2016). This is not to say that no reform attempts have been made; however, even the reforms that have been embraced over the years have barely touched the surface of the core business of schools or the “grammar of schooling” (Tyack & Cuban, 1995; Tyack & Tobin, 1994; Zhao & Watterston, 2021).

Accordingly, this criticism never stopped, and the call for a change in how schooling works has continued to grow. However, the world seems to have been waiting for an opportunity to help guide this change. Thus, researchers think of the COVID-19 crisis as an opportunity to introduce such change. Meanwhile, this pandemic and its implications have presented educators at all levels with countless challenges as they attempt to transition from face-to-face to digital learning (McQuirter, 2020). The global pause that COVID-19 created has led educators at all levels to rethink how education works and what they can do to improve it.

### Main research question

Based on our research problem, we address the following main question in this research:

- Does the digital education shifting (DES) model significantly affect high school students’ engagement?

### Significance of the study

We present a procedural implementation of a DES model that should result in sustainable, successful, and engaging education. DES will add considerable value to existing digital education practices and may improve gains from this kind of education. In addition, we focus on one of the most important instructional aspects—student engagement in the digital instructional environment—because it plays a significant role in improving the quality of educational outcomes.

### Study objectives

We aimed to achieve the following significant objectives:

1. Boost the effectiveness of DES.
2. Enhance students’ engagement in DES.
3. Examine the effects of the DES model on student engagement.

## Theoretical framework

### COVID-19: Educational change catalyst

When the global COVID-19 pandemic first hit in December 2019, the world was left in shock and disarray, and all aspects of life were abruptly and involuntarily disturbed. Almost all industries, except for online-based ones, struggled to respond and adapt to the new norms created by the outbreak. It is safe to say that this pandemic changed the world as we knew it, leading to the tragic loss of numerous lives around the world. Zhao (2020) eloquently described the impact of this pandemic, stating “It has broken rhythms and routines, shattered patterns and norms, and exposed the best and worst of humanity and human institutions” (p. 29).

The spread of COVID-19 continued, resulting in a dramatic increase in the number of cases worldwide (Di Gennaro et al., 2020; Habes et al., 2020). Various countries enforced lockdowns, time limits on services, and even travel restrictions. Although the lockdowns and social distancing enforcements resulted in positive health outcomes, they also provoked the brisk finish of enlightening associations (De Figueiredo et al., 2020). These cross-country terminations affected more than 80% of young understudies and unfairly impacted research and learning activities. Foundations faced incredible challenges responding to these troubles by isolating learning systems (United Nations Educational, Scientific and Cultural Organization, 2020). The education industry was probably one of the worst affected because millions of schools around the world were forced to close (Marinoni et al., 2020), resulting in vast damage that many believe to be incalculable (Zhao, 2020). Luckily, another platform for learning (i.e., online or virtual learning) was already in place to accommodate for the sudden lockdown. However, the readiness and maturity of such learning platforms varied from place to place.

This brings us back to the pause that occurred due to this disaster. Zhao and Watterston (2021) called for governments and education leaders to rethink almost all areas of education. He argued that areas of education fall under three main categories—what, where, and how—and these areas require rethinking to reform education. The *what* of education in this context refers to the curricula prescribed by educational institutions or educational governing bodies. Students have no say in what they must learn, but rather have to master such curricula. A main criticism in this regard is that the same content does not work for all students. Therefore, teachers must consider what their students need to learn and account for individual differences.

The *how* of education here refers to the pedagogical approach that teachers apply to their students. In this regard, Zhao and Watterston (2021) called for project-based learning, in which knowledge is better sought out when it is relevant to learners’ experiences.

Finally, the *where* of education has traditionally been the classroom. Learning has always been defined by what happens in the classroom, and what happens elsewhere is usually considered mere play (Zhao & Watterston, 2021).

Switching from traditional learning to e-learning was an expected outcome of the COVID-19 crisis. E-learning as we know it is now classified into synchronous and asynchronous (Khalil et al., 2020). *Synchronous e-learning* refers to live communication between an instructor and students in the form of videoconferencing or chatting, for example. On the other hand, *asynchronous e-learning* is not bound by time; it features a lag between posting the educational material and its receipt (Finkelstein, 2006). Given these two methods of e-learning, when the COVID-19 crisis hit, the vast majority of the world’s countries moved all forms of education to online learning. They relied on the efficiency of online instructional methods (Aronoff et al., 2010), but also wanted to account for the challenges that students usually face due to the limited verbal communication in online classrooms. Therefore, online education was mainly synchronous, and teachers and students used videoconferencing to cover the required material. This was essential because students’ readiness for online learning was highly questionable. Therefore, the shift that took place was generally toward synchronous e-learning so as to allow for greater teacher supervision.

This shift triggered the need for a model that governs the way education is transformed from one platform to another. Our proposed model provides a solution that comprehensively tackles all aspects of education reform. In the next section, we explore each element of the model in the literature and highlight its importance. By the end, the reader will realize the importance of each element and how all of them work together to build the DES model.

### The digital education shifting model

During the 2017 International Blended Learning Conference at the Saudi Electronic University in Riyadh, Professor Fayiz Aldhafeeri proposed the DES model to assist educators and policymakers in departing from a limited educational perspective and shift toward a digital one utilizing advanced technologies. DES promotes awareness, acceptance, readiness, and orchestration of global teaching and learning. These changes can result in sustainable, successful, and engaging education (Aldhafeeri, 2021). Aldhafeeri (2021) emphasized the importance of establishing a continuous social connection to enable sustainable and successful education. His strategy relies on several educational theories such as social learning, cognitive load, constructivism, and connectivism.

**Fig. 1:**
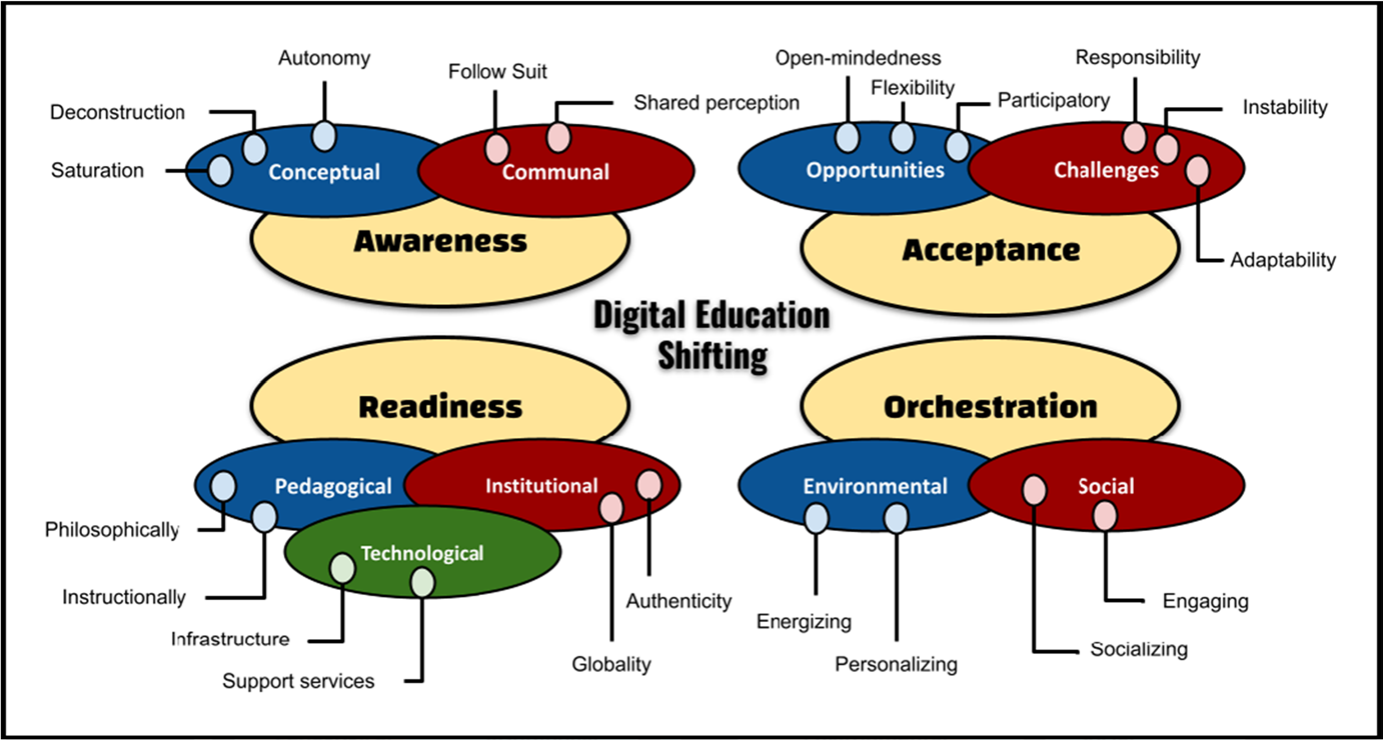
Digital education shifting (DES) model

As shown in Fig. 1, the DES model is divided into four main components: awareness, acceptance, readiness, and orchestration. Each component has certain aspects that must be considered when shifting any educational system toward digital instruction. The model does not focus on a certain mode of educational delivery (i.e., online, blended, or any other form of internet-based distance learning). Instead, the DES model is a strategy that aims to transition away from the traditional educational methods of receiving and teaching information. Also, from the limited view of achievements and quantity, DES is intended to build a sustainable and successful educational system helps stakeholders explore opportunities for using digital technologies that provide engaging learning for teachers, students, and the scientific community. Such technologies may be used in the classroom or online.

To ensure quality and sustainability, the DES model must consider the needs of all members of educational communities, including teachers, parents, learners, curriculum designers, educational supervisors, and relevant staff. Transferring an educational system to one that employs online learning should be done collectively, with the cooperation of all parties involved in the educational process. The implementation of any educational strategy will be ineffective if it is applied independently. For example, if governments authorize teachers to use online learning and train them without increasing the awareness of parents or the educational community, the outcome will be minimally effective (Aldhafeeri & Male, 2020). The DES model assists in the implementation of an effective and beneficial educational system.

### Student engagement

Before indulging online student engagement, the concept of engagement must be distinguished from somewhat similar concepts such as involvement and integration. All of these terms are habitually used interchangeably and may seem similar to many readers, but in fact, they are distinct in several ways (Wolf-Wendel et al., 2009). Astin (2003) defined *involvement* as “the amount of time and physical and psychological energy that the student invests in the learning process” (p. 501). In other words, when students are more involved, they learn and grow exponentially. Involvement can be thought of as an influential approach to enhancing nearly all facets of a student’s cognitive and affective development (Astin, 2003). The concept of involvement should not be confined to academic activities because it could also refer to social activities; nevertheless, a majority of previous studies that have used involvement theory focused mainly on extracurricular involvement (e.g., Pascarella & Terenzini, 1991, 2005, as cited in Wolf-Wendel et al., 2009).

Accordingly, one suggested approach to building a welcoming and supportive campus environment is to involve students socially and academically. Astin (2003) discussed three forms of involvement: involvement with faculty, involvement with peer groups, and academic involvement. Among the three forms of involvement, it appears that involvement with peer groups has the greatest impact on students’ cognitive and affective growth (Astin, 2003). The peer group’s traits and the degree to which a student interacts with that peer group significantly affect all forms of the student’s personal and educational development (Astin, 2003; Weaver & Qi, 2005).

Student engagement in online courses constitutes an essential variable to meet projected learning outcomes, gain a good learning experience, and eventually thrive in these kinds of courses (Bender, 2005; Dixson, 2010; Richardson & Newby, 2006). If students are disengaged when taking online courses, their overall performance and entire learning experience can suffer. Student engagement is a way to ensure the most optimal result from online courses, and is considered one of the main goals of online learning (Khan et al., 2017). Kuh (2009) defined *student engagement* as “the time and effort students devote to activities that are empirically linked to desired outcomes of college and what institutions do to induce students to participate in these activities” (p. 683). Put differently, student engagement is tied to desired college outcomes and the time that students spend doing their academic work along with other educational activities (Kuh, 2009; Wolf-Wendel et al., 2009).

This was the point of view regarding student engagement in traditional classrooms, but is engagement in online learning the same? Research into online learning has mainly focused on the effectiveness of learning (Afrouz & Crisp, 2020; Chauhan, 2017; Sharma et al., 2020), the impact of social aspects in online platforms (Richardson et al., 2017), overall student satisfaction (Gray & DiLoreto, 2016), and so on. However, student engagement in online learning is defined as “the mobilization of cognitive, affective, and motivational strategies for interpretive transactions” (Bangert-Drowns & Pyke, 2001, p. 215) that occurs during learning activities and interactions between students and instructors (Lim, 2004). Engagement is a vital factor that helps keep students committed to learning (Dennen et al., 2007; Kehrwald, 2008; Robinson & Hullinger, 2008; Shea et al., 2006; Swan et al., 2000).

Prior research in online student engagement has viewed it consistently in association with the way students are motivated to learn and keep up with the progress of the online material (Subramanian & Budhrani, 2020). Further, engagement in online learning demands learners’ cognitive effort, mindfulness, and complete attention. To guarantee that students are engaged, it is imperative to know more about them and their needs, what engages them, and what benefits them so that online materials can be better designed. Accordingly, a needs-based analysis is a good place to start when designing online content for a more engaging end result.

In addition to its social and academic impact on students, online student engagement must be a priority for online programs. The literature on this topic indicates that students’ retention rates in online courses were reported to be lower than those for face-to-face courses (Carr, 2000). Consequently, colleges and universities are searching for all conceivable solutions that might assist in increasing retention rates. Luckily, a number of theoretical models have explored student motivation and persistence and showed a positive correlation between retention rates on one hand and academic and social engagement on the other (e.g., Braxton et al., 2004; Hossler & Bean, 1990; Tinto, 1993). In other words, when students are more engaged, retention rates are expected to improve. In this manner, students benefit from being engaged in their online experience, and colleges and universities can also make progress by increasing online students’ retention rates.

### Student experience

Another aspect of online learning is students’ experience in the virtual environment and how important it is for them to learn and thrive (Henry, 2018). Students’ experiences can be thought of as the defining characteristics of online learning and how effective and useful online learning could be. In this regard, students’ experiences are strictly aligned with the level of student satisfaction. In his article, Strachota (2003) claimed that students’ satisfaction in online learning is measured using five typologies of online interaction: learner–content interaction, learner–instructor interaction, learner–learner interaction, computer efficacy, and general satisfaction. Each typology is regarded as a proxy or a factor of measuring student satisfaction in an online environment.

A study that collected and examined data from India and South Korea found that specific variables, such as course facilitation, class interaction, faculty knowledge, motivation, and course structure, certainly influence students’ satisfaction and learning outcomes (Baber, 2020). Harsasi and Sutawijaya (2018) also argued that satisfaction is an individual perception that could be perceived as “how well a learning environment supports academic success” and how well “appropriately challenging instructional methods are serving to trigger students’ thinking and learning” (p. 91). The authors inferred that the most significant elements of student satisfaction in online courses are centered on students and instructors and their roles in learning (Harsasi & Sutawijaya, 2018).

In their study, Gray and DiLoreto (2016) suggested that a significantly strong relationship exists between course structures and student satisfaction in online learning. Further, they also demonstrated that the presence of an instructor had a significant relationship with student satisfaction, but that student interaction, on the other hand, did not display a significant relationship. Notably, the study results exhibited a strong correlation between student engagement and student satisfaction in online programs (Gray & DiLoreto, 2016). In addition, Ng and Baharom (2018) suggested another variable that might contribute to student satisfaction online—students’ self-efficacy of internet and computer use, which is related in part with their readiness for online learning.

### Importance of students’ engagement

In online learning, students typically remain distanced from one another and the instructor, and that disturbs and impacts the overall learning experience (McBrien et al., 2009). Students experience the distance in a way that detaches them from any kind of personal and/or social interaction that might have taken place in a conventional face-to-face classroom. The physical removal from the course instructor and other learners has an impact on students’ engagement as well. In fact, Moore’s theory of transactional distance explicitly argues that distance is a pedagogical phenomenon. Put differently, when learners experience a sense of distance, that feeling extends beyond merely being detached geographically and is actually centered on student engagement in the learning equation (Moore, 1993), which is the issue with online learning, where students are detached from their instructors and colleagues.

Primarily, Moore’s theory of transactional distance includes three elements: dialogue, structure, and learner autonomy. As explained earlier, dialogue interconnects between student–instructor, student–student, and student–content interactions (Ertmer et al., 2011; Lim, 2004; McBrien et al., 2009; Zhu, 2006). The term *structure* in this context refers to the way the content of a course is designed and delivered. In this respect, the instructor indeed plays an indispensable role in formulating purposeful and engaging course designs that endorse interaction, participation, and communication in online learning (McBrien et al., 2009; Robinson & Hullinger, 2008). Consequently, student engagement must be a priority for all stakeholders in online learning from the very early stages of establishing any online content. However, adequate planning must be carried out prior to the implementation of a course so that the design and material are executed effectively and purposefully.

Learners’ autonomy, on the other hand, is embodied by how each student perceives their independent and interdependent participation in the course, and it is directly related to their attitude toward learning (Lim, 2004; McBrien et al., 2009). Online learning is commonly perceived as a student-oriented environment, and students have the ability to decide when to participate and what type of activity they want to participate in. Basically, they have the power to decide when they want to act, where they want to participate, and what they want to do. This is also related to the importance of designing meaningful tasks that appeal to the students and helping engage them in the course content more often than not. Along with that, students’ personal attitudes toward learning and their benefits from these useful activities help engage them even more. Nonetheless, if a student demonstrates reluctance to participate and an adverse attitude toward learning, this might actually lead to disengagement.

### Student disengagement

As opposed to student engagement, students might also be disengaged from learning in the virtual environment. An issue that might lead to disengagement is the assumption that learners already have the appropriate attitude to learn. While some students do have the appropriate attitude toward learning, some do not; they simply browse the screen while participating in online courses and expect the learning to miraculously happen. Other students may also browse an online course merely to get the right results/answers without any intention to learn. Many other scenarios might take place in such a distance learning environment; therefore, researchers have tried to address this problem through the use and design of interactive online activities in which students have to interact by answering questions continuously as a form of formative assessment.

Generally speaking, authentic online activities are capable of motivating students to participate by facilitating student engagement with the instructional message of the online course material (Lim, 2004). Thus, providing them a reason as to why they are learning certain materials will give them the motivation they need for initial perseverance in any online course. When students are aware of what and why they are learning, they are more likely to get engaged in the learning process (Lim, 2004).

To further engage students in online courses, research has suggested that the communicative approach should be used to facilitate learning. This approach calls for using learning activities that are based on real-life situations so that students can make better sense of what they are learning and become more engaged in the learning process (Lim, 2004). As a matter of fact, the communicative approach has proved to be very effective in many areas of knowledge, such as language learning, science, medicine, math, and so on, due to the way it approaches students’ understanding through the use of analogies of familiar concepts and real-life experiences.

### Principles of good practice

Another attempt to engage students in an online course is observable in a study conducted by Silverstone and Keeler (2013), who attempted to update an undergraduate marketing online course that includes the seven key principles of effective teaching identified by Graham et al. (2001). The seven principles are listed below:

- Principle 1: Good practice encourages student–faculty contact
- Principle 2: Good practice encourages cooperation among students
- Principle 3: Good practice encourages active learning
- Principle 4: Good practice gives prompt feedback
- Principle 5: Good practice emphasizes time spent on a task
- Principle 6: Good practice communicates high expectations
- Principle 7: Good practice respects diverse talents and ways of learning

These principles are considered a landmark in the design of online courses (Silverstone & Keeler, 2013). The first principle, encouraging student–faculty contact, dictates that instructors should provide clear guidelines for interaction with their students. Ten days before the beginning of a class, a course syllabus and a welcome letter from the instructor providing their contact information are sent to all students via e-mail. Taking initiative in communication is the instructor’s responsibility because it allows them to set the rules and policies of the course as well as address the various means of communication among the students and with the instructor.

In general, student–faculty interaction via e-mails, discussion boards, assignment comments, and so on are all helpful to engage students and help them adjust to the online course. Since these students do not physically meet with the instructor, all forms of communication are recommended to keep them mentally and psychologically linked to the course. More responsibility lies with the instructor in this regard to encourage communication with the students, but students are also highly encouraged to keep an active connection with their instructor (Silverstone & Keeler, 2013).

The second principle promotes the idea that “well-designed discussion assignments facilitate meaningful cooperation among students” (Silverstone & Keeler, 2013, p. 17). Therefore, the instructor could utilize online discussion boards, which constitute a very valuable tool of engagement among students (Silverstone & Keeler, 2013). In setting up these discussion boards, some guidelines must exist. For example, all the students must submit a minimum number of posts per topic and respond constructively to a minimum number of their peers’ posts. In assigning these rules, discussion boards will turn to a lively discussion of the topic at hand and achieve the maximum benefit. In addition, the topic of discussion must be relevant to the course material, and required readings and should guide the students in investigating various aspects of the topic so they can maximize their understanding.

In addition to discussion boards, Silverstone and Keeler (2013) also recommended that instructors use chat sessions fairly and regularly due to their interactive nature and lively content. To employ chat sessions effectively, the instructor needs to prepare an agenda for the chat and submit it to the students so that the time is used properly and systematically, avoiding random discussions. These chat sessions can be voice or video sessions, and there are a number of conferencing applications that can be used to start a live chat session, such as Class Live Pro, which features “shared interactive whiteboard, application sharing, live webcam video, breakout rooms, chat, graphing calculator, PowerPoint import, content import, record and playback feature, polling, synchronized web browsing, and the archiving of sessions” (Silverstone & Keeler, 2013, p. 18). All of these features are embedded within Class Live Pro and many other conferencing programs to facilitate student–instructor interaction and simulate real conversations.

The third principle highlights active learning, which refers to the process by which students take part in activities that promote analysis, synthesis, and evaluation of class content. Put differently, students submit periodical assignments and class projects and receive feedback on them from the instructor. Expectations for such assignments should be stated ahead of time, and the teacher should make the tasks challenging yet manageable. It is also important to note here that written input is all that the instructor will get from students; therefore, their writing should be good enough to convey the requirements of the assignments and class projects.

Discussion board postings, weekly synchronous chat sessions, and weekly assignments are all valuable assets to engaging students (Silverstone & Keeler, 2013). In addition to those, the fourth principle urges instructors to provide prompt feedback. There are two types of feedback: information and acknowledgement (Silverstone & Keeler, 2013). This principle suggests that teachers provide immediate feedback that satisfies the students’ concerns. Online students may feel isolated and distant, and they tend to expect immediate feedback regarding their questions and concerns; that is why managing online classes is different from face-to-face ones. Cull (2010) clarified this point by stating that “in a face-to-face classroom, instructors have an opportunity to interact with the students, respond immediately and be aware of visual and nonverbal cues from students that can indicate they are disengaged, frustrated or unenthusiastic” (as cited in Silverstone & Keeler, 2013, p. 21). Thus, a successful online instructor would solve this issue by providing students with prompt information and acknowledgement feedback. The value of this feedback is tremendous for students, as it helps them stay on track and guided throughout the course. As a best practice, Silverstone and Keeler (2013) suggested that an instructor should provide feedback within 24 h.

The fifth principle, emphasizing time on task, refers to the fact that online courses need deadlines (Silverstone & Keeler, 2013). Students usually have the preconception that online courses are less demanding than face-to-face ones. As a matter of fact, some students enroll in online courses because they believe they are easier and require less time, which is not true at all. Therefore, students are prone to disengagement before an online class even starts, and that makes the instructor’s task much more difficult because they have to dismantle that perception from the students’ minds. As a result, providing clear instructions with regard to all requirements and deadlines gives the online course a timely framework and structure, and it makes the students aware of the instructor’s expectations of the instructor and the course as a whole (Silverstone & Keeler, 2013). In addition, sending reminders to the students before the due date of any assignment is a good practice and helps them stay on track. Finally, in terms of online quizzes and tests, students should be made aware of when these quizzes and tests will be available and when they will go inactive; sending reminders of these due dates and times is also recommended.

Moving to the sixth principle, communicating high expectations, “challenging tasks, sample cases, and praise for quality work communicate high expectations” (Silverstone & Keeler, 2013, p. 21). Instructors need to encourage their students and help them push their limits. The learning experience becomes fun when students feel they are challenged, and the outcome of those challenges is a product they can be proud of. Accordingly, the instructor needs to communicate high expectations to the students in all course requirements. For example, the instructor should ask students to complete their class assignments to the best of their ability and praise them when they are successful. When giving feedback, the instructor should always start with positive feedback and then highlight what could improve the quality of the students’ work. In general, assignments will be graded based on a rubric that the students use as a guide to write their assignments. An exceptional case here is the final project, in which the instructor chooses to provide the students with an example project to indirectly communicate high expectations.

Finally, the seventh principle completes the picture by indicating that “good practice respects diverse talents and ways of learning” (Silverstone & Keeler, 2013, p. 22). Thus, calling for quality is an important thing, but the instructor should also consider individual differences and respect them (Silverstone & Keeler, 2013). Students learn differently and at various speeds, and instructors should first do a diagnosis of their students to identify individual differences among them. In fact, some students might need significantly more time than others to understand concepts and adjust to new learning environments. Therefore, instructors are encouraged to take the initiative and communicate with the students they think need extra support as well as provide assistance in a way that further engages them. Additionally, the instructor must keep the content challenging for the other students who seem to progress quickly in the course, so they do not become bored and disengage.

All in all, these seven principles of good practice seem to provide a thorough model of successful online teaching experience and ensure that teachers do a good job instructing and engaging students. These principles apply to the online experience from various perspectives and can encourage both students and teachers to follow good practices so they can make the learning experience worthwhile.

## Methodology

### Research context

Although the DES model is intended to be used worldwide, the context of this study was the Kuwaiti school system. Overall, six school districts in Kuwait are governed directly by the Kuwaiti Ministry of Education. As is the case with many Arab and Gulf countries, K–12 education is separated, so girls and boys do not attend the same schools. High school in Kuwait goes from Grades 10 to 12. In Grades 11 and 12, students are required to choose or be assigned to one of two tracks: science or liberal arts. The science track focuses on subjects such as math, physics, chemistry, and biology; the liberal arts track focuses on subjects such as history, geography, and philosophy.

When the COVID-19 crisis hit, the Kuwaiti government, like many other countries around the world, decided to move all forms of education online. In-person education was postponed as a security measure to reduce the spread of the virus. This is important for the reader to realize that all forms of training and meetings held for the purpose of this study were initiated online.

### Design of the study

The current study is intended to examine the effect of the DES model on students’ engagement. Thus, we employed an experimental approach for the purpose of serving this study’s objectives, which we carried out in various educational contexts. Moreover, we adopted a newly designed teachers’ training course and a students’ engagement self-checklist instrument, to gather the research data.

### Procedures

We conducted the current study in the Kuwaiti school system into two phases. We started the first phase by preparing a training course geared toward preparing schoolteachers to implement the DES model in practice. Prior to its implementation, a panel of experts reviewed and approved the content of the course, which focuses on the DES model’s main and minor components. Following that, we trained teachers in one of the chosen schools using the content and instructions of the training course. Teachers in the other school did not receive any kind of training from us. After the training was over, we moved to the second phase by collecting data from students in both schools to measure their level of engagement. Students in the school where the teachers received the training were considered the experimental group, and those from the other school were considered the control group. We used data from this phase to answer the research question.

### Participants

We drew the sample from two female high schools located in the Al-Jahra school district in Kuwait. High schools in Kuwait start at Grade 10. Starting in Grade 11, Kuwaiti students are assigned to one of two tracks (i.e., science or liberal arts). The student population in the experimental school was 746 female students, and the control school had 667 female students. All participants were traditional female high school students ranging from 15 to 18 years old. Of course, the students were all Kuwaiti nationals whose first language is Arabic. The choice of gender was due to two reasons. The first refers to gender segregation into male and female schools in Kuwait public schools; one of these researchers who collected the data is a female, so she was only allowed to access female schools, especially during the COVID-19 restrictions. Another reason is due to the historical misconceptions about females’ education and the need to achieve the objectives of the current study with reference to their abilities to learn in the DES environment.

### Instrumentation and data collection

We created a self-checklist tool measuring the level of student engagement in the digital environment. This instrument was mainly inspired by Kandiko and Matos (2013) and Delfino (2019). We also presented items for this instrument to a panel of experts for review. Some items were deleted, some were modified, and some were placed in a different order. The final version contained 28 items that we measured using a Likert-type scale. Due to the potential language barriers, we also translated the instrument into Arabic using a native translator. We distributed two versions of the tools: one for the experimental group and another for the control group. We then collected data using Qualtrics Surveys for both groups.

### Analysis and study variables

When assigning the groups, some main variables such as age, gender, and socioeconomic status were taken into consideration equally when assigning the experimental and control groups.

Analysis of the second phase of the research was based on a quantitative approach. The experimental nature of this phase of the study required identifying whether a difference existed between the two groups. Because there are only two groups (i.e., experimental and control), the appropriate method of analysis in this context would be a *t*-test. However, because the participants were from two different schools, a nested model or a multilevel model might yield better results. Accordingly, we examined the intraclass correlation coefficient prior to the analysis to determine the most appropriate method of analysis. Besides the DES variables, we also accounted for other variables, namely age, gender, educational level, and study track.

## Findings

### Data cleaning

We collected data online via Qualtrics Surveys, and the raw total number of respondents reached 245—115 in the experimental group and 130 in the control group. However, upon examining the responses individually, 26 respondents from the experimental group merely responded to the screening questions and exited the survey. In terms of the duration of time spent on the survey, this group of participants spent less than 2 min on the survey on average. These were considered incomplete responses and were excluded. This brought the final count for the experimental group to 89 participants. Similarly, the control group had 12 incomplete responses following the same pattern as the experimental group. These cases were removed as well. This brought the final count for the control group to *n* = 188, and the overall sample size was *N* = 207 complete responses.

To avoid analytical challenges due to missing data, we decided to eliminate partial responses and only include complete responses in our analysis. Upon cleaning up the data, we looked into any potential outliers that could influence the results and introduce bias (Osborne & Overbay, 2004). By definition, an *outlier* is a given observation whose value is fairly far from other data points (Enderlein, 1987). Fortunately, variables in our data primarily used a Likert scale with a range of 1 to 5. Therefore, we checked the minimum and maximum values to ensure no values exceeded this scale due to typos. All data were coded automatically using Qualtrics Surveys during collection, so no extreme values were recorded.

### Descriptive statistics

Before analysis, a common practice is to get acquainted with the data and acquire a first impression of the sample. Our participants ranged in age from 15 to 18 years old. Table 1 shows that 31.4% of the participants reported their age as 18 years old. The remainder of the participants were 15, 16, or 17 years of age, which constituted 18.8%, 24.6%, and 25.1% of the sample population, respectively. These are the ages of traditional high school students, and our sample represents them well. There were more participants in the higher age ranges, meaning an easy acceptability of the research. Additionally, these individuals comprehended the goal pf the research and cooperated to ease the research.

**Table 1:** Participant Ages

Age		Frequency	Percent	Valid Percentage	Cumulative Percentage
Valid	15	39	18.8	18.8	18.8
	16	51	24.6	24.6	43.5
	17	52	25.1	25.1	68.6
	18	65	31.4	31.4	100.0
	Total	207	100.0	100.0	

Similarly, participants came from high schools; i.e., Grades 10 through 12. Around 41% of the participants were 12th-grade students, 28% were 11th-grade students, and 31% were 10th-grade students (Table 2). About 59% of the 11th-graders were in the science track, while 41% reported being in the liberal arts track (see Table 3). Similarly, 36% of the students in Grade 12 reported being in the science track, while the remaining 64% were in the liberal arts track. Overall, about 45% of all participants were in the science track, while 55% were in the liberal arts track. This distribution of participants across the demographic variables seems justifiable, as the sample represents the schools that they were sampled from.

**Table 2:** Grade Levels

Grade		Frequency	Percentage	Valid Percentage	Cumulative Percentage
Valid	10th grade	64	30.9	30.9	30.9
	11th grade	59	28.5	28.5	59.4
	12th grade	84	40.6	40.6	100.0
	Total	207	100.0	100.0

**Table 3:** Grade * Track Cross-Tabulation

			Track		Total
			Science	Liberal Arts	
Grade	11	Count	35	24	59
		Within Grade	59.3%	40.7%	100.0%
		Within Track	53.8%	30.8%	41.3%
		Percentage of Total	24.5%	16.8%	41.3%
	12	Count	30	54	84
		Within Grade	35.7%	64.3%	100.0%
		Within Track	46.2%	69.2%	58.7%
		Percentage of Total	21.0%	37.8%	58.7%
Total		Count	65	78	143
		Within Grade	45.5%	54.5%	100.0%
		Within Track	100.0%	100.0%	100.0%
		Percentage of Total	45.5%	54.5%	100.0%

To investigate the sample further, Table 3 shows that around 59% of 11th graders were in the science track, while 41% reported being in the liberal arts track. Similarly, 36% of the students in Grade 12 reported being in the science track, while the remainder (64%) were in the liberal arts track. Overall, about 45% of the participants were in the science track, while 55% were in the liberal arts track. This distribution of participants across the demographic variables seems justifiable, as the sample represents the schools that they were sampled from.

The main research question we asked was whether DES significantly affects students’ engagement. To answer that question, we conducted a series of one-way ANOVAs (see Table 5). As expected, statistically significant differences emerged between the two groups across all factors of engagement, all of them in favor of the experimental group (see Table 4). For example, academic engagement significantly affected the experimental and control groups at the *p* < 0.05 level (*F*[1, 205] = 17.22, *p* < 0.01). Behavioral engagement also significantly affected the experimental and control groups at the *p* < 0.05 level (*F*[1, 205] = 16.84, *p* < 0.01). The same could also be said about cognitive, affective, internal, and observable forms of engagement.

**Table 4:** Descriptive Statistics

		*n*	*M*	*SD*	*SE*	95% CI for *M*		Min	Max
						LL	UL		
Academic Engagement	Control	89	2.03	.649	.069	1.902	2.175	1.00	4.14
	Experimental	118	2.49	.862	.079	2.334	2.649	1.00	5.00
	Total	207	2.30	.808	.056	2.186	2.407	1.00	5.00
Behavioral Engagement	Control	89	1.79	.470	.049	1.693	1.891	1.00	3.33
	Experimental	118	2.21	.867	.079	2.051	2.367	1.00	5.00
	Total	207	2.03	.751	.052	1.927	2.133	1.00	5.00
Cognitive Engagement	Control	89	1.85	.604	.064	1.717	1.972	1.00	3.71
	Experimental	118	2.35	.972	.089	2.178	2.532	1.00	5.00
	Total	207	2.14	.869	.060	2.016	2.254	1.00	5.00
Affective Engagement	Control	89	1.95	.687	.071	1.809	2.098	1.00	4.50
	Experimental	118	2.48	1.024	.094	2.289	2.664	1.00	5.00
	Total	207	2.25	.929	.064	2.124	2.379	1.00	5.00
Internal Engagement	Control	89	1.90	.593	.063	1.778	2.027	1.00	4.13
	Experimental	118	2.42	.969	.089	2.243	2.597	1.00	5.00
	Total	207	2.197	.86594	.06019	2.079	2.316	1.00	5.00
Observable Engagement	Control	89	1.925	.49865	.05286	1.819	2.029	1.00	3.46
	Experimental	118	2.361	.80612	.07421	2.214	2.508	1.00	5.00
	Total	207	2.173	.72263	.05023	2.075	2.272	1.00	5.00

## Results

The results in academic engagement evidence means from the experimental procedures and those of the control experiment were different by 0.49, as shown in Table 4. The same differences were evident throughout all the statistical analysis throughout the study. This means that the control results and those of the experiment differed by some values. The difference could be linked to internal validities leading to shifts in the responses during the research. Behavioral and cognitive engagement had the lowest results in the control and experimental calculations. This could point to differences in skill sets among the individuals leading to the low cumulative results.

The standard deviations between the control and the experimental values differed by wide margins, corresponding to a proportionate margin from the mean values. The same is observed in Table 5, including more data on effective, internal, and observable engagement.

**Table 5:** ANOVA

		Sum of Squares	*df*	*M* Square	*F*	*P-value*
Academic Engagement	Between Groups	10.411	1	10.411	17.216	.000
	Within Groups	123.972	205	.605		
	Total	134.383	206			
Behavioral Engagement	Between Groups	8.818	1	8.818	16.838	.000
	Within Groups	107.359	205	.524		
	Total	116.177	206			
Cognitive Engagement	Between Groups	13.218	1	13.218	19.003	.000
	Within Groups	142.587	205	.696		
	Total	155.804	206			
Affective Engagement	Between Groups	13.880	1	13.880	17.322	.000
	Within Groups	164.260	205	.801		
	Total	178.140	206			
Internal Engagement	Between Groups	13.569	1	13.569	19.741	.000
	Within Groups	140.901	205	.687		
	Total	154.470	206			
Observable Engagement	Between Groups	9.660	1	9.660	20.224	.000
	Within Groups	97.911	205	.478		
	Total	107.571	206			

## Discussion

In our study, students in the experimental group recorded higher levels of student engagement than those in the control group. This suggests that such a difference could be attributed to the quasi-experimental design that allowed teachers in the experimental group to be trained according to the DES model. Put differently, it is safe to say that the content of the DES model was effective to the point that it made a difference in students’ levels of engagement. We hypothesized that when the DES model was implemented appropriately, student engagement would be positively impacted, and this appeared to hold true. Therefore, adopting the DES model in any education shift is highly advisable, as it yields positive outcomes for learners and their experiences.

Adopting such a model will improve students’ engagement levels. Consequently, our.

results conform with the literature; engagement boosts students’ satisfaction and improves their learning experience (Gómez-Rey et al., 2016; Gray & DiLoreto, 2016; Henry, 2018). In addition, student satisfaction and engagement in online learning are discussed in the literature in regard to students’ motivation (Subramanian & Budhrani, 2020).

### Implications and recommendations

The DES model proved to significantly impact students’ engagement. Although the teachers in the experimental group received relatively brief training, the DES model still yielded results. Accordingly, we encourage extending training sessions to cover all teachers during an educational shift. Doing so would ensure a smooth and successful transition that would ultimately boost the success of an educational program. In addition, we highly recommend holding workshops for all education stakeholders to educate them about the DES model and how it facilitates shifts. Among these stakeholders, special attention should be given to those who are part of policy councils so that the DES model can be given more attention and potential endorsement.

### Limitations of the study

Any given study may be deemed to have a number of limitations, for perfection is a far-fetched goal. Realizing one’s shortcomings in a research endeavor is an important aspect of research, as it allows researchers to work around issues instead of ignoring them. In the current study, we identified some limitations that should be taken into consideration when interpreting or generalizing our results. First, our study sample was taken entirely from female-only schools. Therefore, these results should be used with caution, especially when generalizing to male or coed schools. Additionally, the entire sample was taken from Kuwait, so global generalization of our results may not be rational. Finally, although the sample size was large enough to yield statistically significant results, we believe that it was not sufficient to generalize results on the global level.

### Future research

Replicating this study across a more representative sample would yield interesting results. From the perspective of time, future studies should look into the amount of time required to train teachers on the DES model to yield significant results. Finally, investigating the impact of the DES model on other educational issues, such as readiness and awareness for both students and teachers, should also be studied.
